# Local Use of Hydrogel with Amiodarone in Cardiac Surgery: Experiment and Translation to the Clinic

**DOI:** 10.3390/gels7010029

**Published:** 2021-03-10

**Authors:** Vladimir Shvartz, Teymuraz Kanametov, Maria Sokolskaya, Andrey Petrosyan, Tatyana Le, Olga Bockeria, Leo Bockeria

**Affiliations:** Bakulev National Medical Research Center for Cardiovascular Surgery, Department of Surgical Treatment for Interactive Pathology, Rublevskoe shosse 135, 121552 Moscow, Russia; tima586@mail.ru (T.K.); sokolskayam@mail.ru (M.S.); adpetrosyan@bakulev.ru (A.P.); tanya_co@mail.ru (T.L.); olbockeria@bakulev.ru (O.B.); leoan@bakulev.ru (L.B.)

**Keywords:** amiodarone-releasing hydrogel, heart rate, postoperative atrial fibrillation, prevention

## Abstract

The objective of this study was to study the use of the hydrogel biopolymer based on sodium alginate (“Colegel”) with a drug substance—amiodarone—for the prevention of postoperative atrial fibrillation (POAF) in cardiac surgery. The experimental part of the study was performed on 46 rabbits. Five groups were formed: in the first group, the dose of amiodarone in hydrogel was 1 mg; in the second group—3 mg; in the third group—6 mg; in the fourth group, hydrogel was used without amiodarone; in the fifth group, 60 mg amiodarone was administered intravenously. The animals from each group were removed from the experiment for the pathomorphological study of the heart after 3, 7 and 14 days. The studied endpoints were: the heart rate control; the development of the blockades of the conduction system of the heart; and the development of inflammation according to laboratory pathomorphological studies. The translational clinical part involved a randomized clinical trial which included 60 patients, with an average age of 62 ± 8.5 years. All patients were randomized into two groups: the study group (*n* = 30, with the application of amiodarone hydrogel) and the control group (*n* = 30, without the application of amiodarone hydrogel). The dose of amiodarone in the hydrogel material was 60 mg for all patients. The heart rhythm was monitored during 5 days. The primary endpoint was the development of POAF. Secondary endpoints were: the dynamics of heart rate; the duration of the QT and PQ intervals; the development of blockades of the cardiac conduction system; as well as the dynamics of AST and ALT. According to the results of the experimental part, it was found that the method of the local epicardial delivery of amiodarone by the hydrogel material was safe. Hydrogel with amiodarone is effective for reducing the heart rate in the animal experiment in comparison to the control group and the group with the intravenous administration of the drug. The optimal dose of amiodarone in hydrogel was 1 mg per 1 kg. According to the results of the clinical part, it was found that the method of the local epicardial delivery of amiodarone as a hydrogel material proved its safety. Hydrogel with amiodarone at a dose of 60 mg was effective in preventing POAF in patients after coronary artery bypass grafting (CABG) operations in comparison to the control group (*p* < 0.001). The age and procedure of application of the amiodarone gel were significantly associated with POAF (*p* = 0.009 and *p* = 0.011, respectively). The use of hydrogel with amiodarone reduced the probability of developing POAF 18.9-fold. The method of the local epicardial delivery of amiodarone in the form of a hydrogel material is safe. The use of hydrogel with amiodarone after CABG reduced the probability of developing POAF.

## 1. Introduction

Atrial fibrillation (AF) is the most common complication after open cardiac surgery. It has been proven to be associated with an increase in the frequency of hospital strokes, myocardial infarction, and mortality, which increase the length of hospital stay and economic costs [1,2]. Usually, postoperative atrial fibrillation (POAF) is observed within the first week after surgery or even later [3,4]. POAF tends to occur within 2 to 6 days after surgery [4]. The peak incidence for the development of POAF is between the second and third days after surgery [1], with frequent relapses during the first week [5]. The majority of cases of POAF (more than 90%) are resolved within 4 to 6 weeks after surgery [6].

According to current literature data, the frequency of AF after open heart surgery is approximately 10–65% and depends on the type of intervention: coronary artery bypass grafting (CABG), single-valve surgery or multi-valvular correction as well as the combination of interventions [1,5]. The frequency of the occurrence of atrial fibrillation detection significantly depends on diagnostic methods. Daily electrocardiographic (ECG) monitoring is characterized by a higher detection rate than routine 12-lead ECG [7]. The most accurate diagnostic value can be obtained by the multi-day (up to 14 days) ECG monitoring systems that are currently available on the market [8].

Amiodarone is the most effective antiarrhythmic drug for the treatment and prevention of AF. In the ACC/AHA, ESC, and AATS guidelines [9,10], amiodarone is a IIa class recommendation of evidence level A for the prevention of POAF. However, its application in clinical practice is often limited by its association with a high frequency of extra-cardiac side effects. Usually, these are caused by the effects of systemic saturation of the body with the drug.

The use of the local application of amiodarone directly on the heart is a promising trend which reduces its systemic side effects. The use of biotechnological polymer systems for creating materials with a prolonged release of a drug substance and their local application on the heart are a new and promising trend in cardiac surgery.

Our aim was to develop a methodology for locally using amiodarone in the prevention of AF after open-heart cardiac surgery.

This study included two parts: the experimental and the clinical. At the first stage, we studied the safety of using the hydrogel material “Colegel” with amiodarone and the efficiency of various doses of amiodarone in this gel in reducing the heart rate. At the second stage, a randomized clinical study of the use of the hydrogel material “Colegel” with amiodarone in patients during CABG was conducted.

## 2. Results and Discussion

### 2.1. Results of the Experimental Part

Table 1 shows the initial instrumental and laboratory characteristics of the groups. Initially, the parameters did not differ. Table 2 shows the results of heart rate at control points in each group.

**Figure 1:**
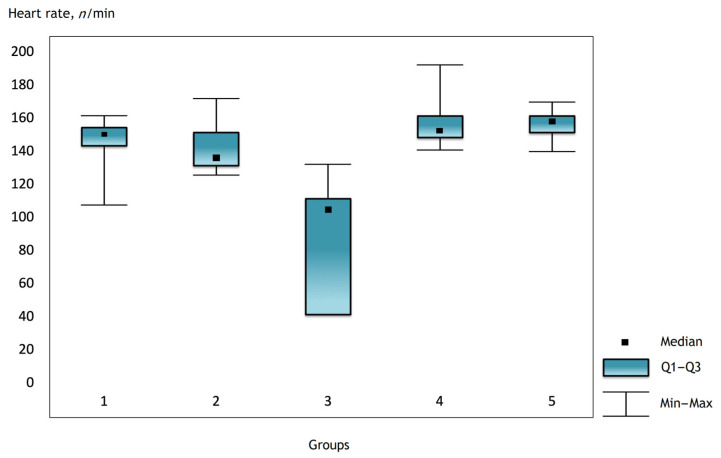
The heart rate in the groups 2 h after surgery.

**Figure 2:**
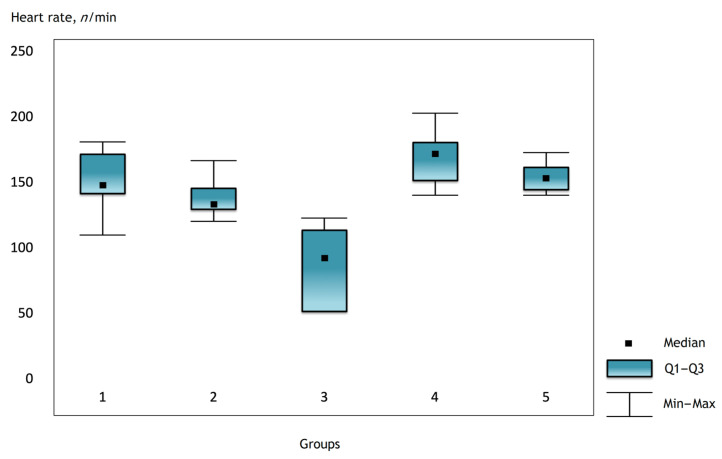
Heart rate in the groups on the first day after surgery.

**Figure 3:**
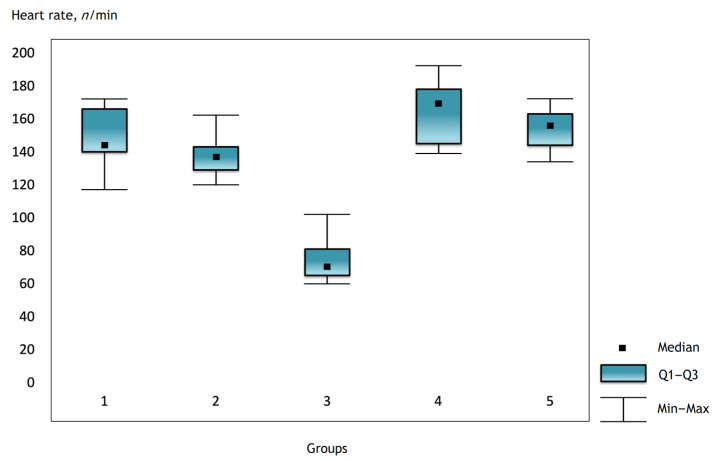
Heart rate in the groups on the third day after surgery.

It was noted that before the operation and after the administration of atropine, the heart rate was comparable in the groups, but there were statistically significant differences in the groups immediately after the operation and further in the dynamics (Figure 1, Figure 2 and Figure 3).

Conduction disorders after the intervention were detected in groups 3 and 5—in 71 and 20% of cases, respectively. Additionally, there was a statistically significant increase in the level of liver enzymes (AST, ALT) in these groups (Table 3).

**Figure 4:**
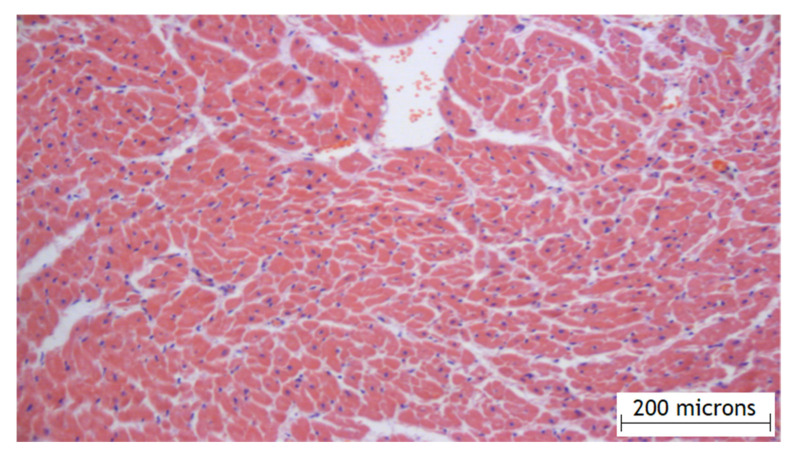
Histology of the right atrial myocardium after the application of the hydrogel with amiodarone.

**Figure 5:**
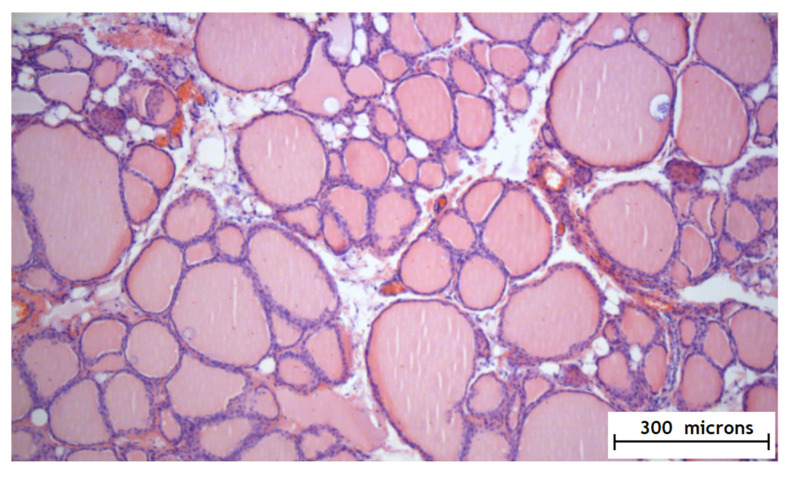
Histology of the thyroid gland after the application of the hydrogel with amiodarone.

According to the results of the experimental part, it was found that the method of the local epicardial delivery of amiodarone in the form of hydrogel material is safe: no tissue damage, no changes in their structure, and no signs of inflammation were detected according to histological studies and there were no ischemic and contractural injuries (histology Figure 4 and Figure 5).

Hydrogel with amiodarone was effective for reducing heart rate in the animal experiment compared to the control group (without amiodarone) and the group with the intravenous administration of the drug. The optimal dose of amiodarone was 3 mg (group 2). At a dose of 1 mg of amiodarone gel, the efficiency in reducing the heart rate was not statistically significant (*p* = 0.244); and at the dose of 6 mg of amiodarone, cardiac conduction disorders were detected (71%), as well as increased levels of liver enzymes.

Thus, taking into account the average animal weight of 3–4 kg, we found that the optimal dosage of amiodarone in a hydrogel is 1 mg per 1 kg of weight [11].

### 2.2. Results of the Clinical Part

After the CABG, the rhythm was monitored for 7 days using Holter ECG monitoring. The development of AF was defined as an episode of AF lasting more than 5 min.

The toxic and infectious effects of the hydrogel were also evaluated on first and fifth days after surgery by analyzing the level of white blood cells and white blood cell formula, as well as analyzing their level in connection with AF episodes.

Table 4 shows the initial clinical, laboratory and instrumental parameters. The groups had no significant differences. Only one parameter (left atrial volume) was significantly different. However, the difference was in the direction of increasing the probability of developing AF in the study group (the volume of the left atrium was larger in the group with the application of amiodarone gel), but not in the control group. There were also no statistically significant differences in the intraoperative data.

Statistically significant differences between the groups were found in the frequency of POAF: in the study group, AF was detected in one patient, which represented 3.3%; in the control group, the frequency of POAF was 37% (*p* < 0.001). The level of white blood cells on the first day after surgery in both groups was comparable and did not differ significantly. In addition, there were no statistically significant differences in all laboratory parameters on the fifth day: white blood cells, glucose, lactate, creatinine. When analyzing the ECG on the fifth day, there were statistically significant differences relative to the PQ interval (*p* = 0.002). There were no significant changes in the duration of QRS and QT intervals (Table 5).

The average heart rate on the fifth day after surgery in the study group was lower than in the control group (*p* < 0.001) based on the Holter ECG monitoring data. There were also significant differences in the minimum heart rate per day (*p* = 0.008).

The risk of POAF was calculated using the Cox regression model (Table 6). Only age (*p* = 0.009) and the application of hydrogel with amiodarone (*p* = 0.011) showed statistical significance among all clinical, laboratory, and instrumental parameters [12].

The pathogenesis of atrial fibrillation after heart surgery has been studied in numerous trials. It is multifactorial and includes preoperative (age, sex, hypertension, myocardial infarction, etc.), intraoperative (prolonged artificial circulation, cardioplegia, myocardial ischemia, reperfusion syndrome, etc.) and postoperative (infection, inflammation, electrolyte imbalance) predictors [13].

The search for new alternative ways to increase the efficiency and reduce the severity of adverse side effects of drugs has led to an increasing interest in their local use [14,15,16]. Local application methods are widely used in oncology, dermatology, and traumatology. In modern cardiac surgery, this kind of application of drugs is more experimental in nature.

Recently, there has been a division between synthetic and natural polymers according to their primary purpose. Synthetic polymers, especially those of the latest generation, which are highly resistant to various influences (temperature, pH of the medium, mechanical deformations, etc.), are used when these effects occur in the human body. First of all, implants, for which stability over time is one of the main advantages [17].

Natural polymers that biodegrade in the body (biohydrolysis) are used in cases where the biopolymer performs, as a rule, an important function of a temporary treatment depot (matrix), which contains drugs in its structure and releases them in the right place, at the right time, according to the concentration profile necessary for treatment. After performing the function of a delivery vehicle and “container”, the biodegradable biopolymer is removed from the body. Biopolymers have more or less a set of therapeutic properties (biocidal, antioxidant, anti-allergic, etc.). The main advantage of biopolymers over synthetic polymers, in addition to their ability to biodegrade, is the absence of toxicity. This is important in all fields of application, and especially in medicine [18].

We used a hydrogel material “Colegel”, made on the basis of a biopolymer of sodium alginate. Since the hydrogel uses sodium alginate obtained from seaweed as a thickener [1] and is a good source of growth and reproduction of bacteria, it is subject to mandatory radiation sterilization—at a dose of no more than 6 KGy [8], and products made from it are subject to microbiological control.

An important property of this biopolymer is its adhesion to tissues. With this system of local delivery, the concentration of amiodarone in the atrial myocardium remains very high, since the adhesive material helps to preserve the substance in the atrium and does not allow it to “leak” from the atrial tissues. The use of sodium alginate in our study was associated with a lower rate of its swelling and biodegradation and an increase in its presence on the surface of the atria, which allowed us to retain the drug substance longer. Thus, amiodarone was released slowly and smoothly, and the slow biodegradation of sodium alginate allowed this release to be prolonged as long as possible.

The development of sinoatrial and atrioventricular blockades is a possible complication of local amiodarone application in the form of a hydrogel. The correct calculation of the amiodarone dose in the gel helps solve this problem. We determined the optimal dosage of amiodarone in a hydrogel in the experimental part of our study. It was 1 mg per 1 kg of weight. When we used such a concentration of the drug, there was a significant decrease in the average heart rate, without the development of intra-atrial and atrioventricular blockades. We noted a significant increase in the development of cardiac conduction disorders (about 70%), when we raised the concentration of amiodarone to 6 mg. When we reduced the dosage to 1 mg, the effect of amiodarone in reducing heart rate was absent. The hydrogel base had no evident effect the conducting system. We did not conduct a quantitative study of the content of amiodarone in the blood. In the experimental part, we focused on the effects of amiodarone on the cardiac conduction system, heart rhythm and tissue pathomorphology during histological examination.

In the clinical part of the work, we found a high efficiency of using this composition: hydrogel with amiodarone at a dose of 1 mg per 1 kg of patient weight, in the prevention of AF in patients in the early postoperative period after CABG. The age and application of the hydrogel showed statistical significance among all the parameters.

Some studies have shown that the level of white blood cells and neutrophils is a specific independent predictor of POAF in the framework of the hypothesis of an inflammatory nature in the genesis of POAF [19]. In our study, the level of white blood cells was comparable at all control points. This indicates that the hydrogel itself does not have an anti-inflammatory effect, and the efficiency is due to the cellular antiarrhythmic effect of the drug amiodarone when it penetrates the atrial myocardium.

The mechanism of the antiarrhythmic effect of amiodarone in local use is absolutely the same as with systemic use: it increases the duration of the action potential (phase III) and the effective refractory period and reduces the excitability of the myocardium. The penetration of amiodarone contained in the hydrogel into the cell is caused by diffusion mechanisms [20]. The mechanism of gel substance elimination from the pericardial cavity is associated with lymphatic drainage processes [18]. In the clinical part of the study, the amount of amiodarone in the blood was also not evaluated, since the experience of such studies showed that the level of amiodarone in the blood plasma with its local use at the rate of 1 mg per 1 kg remained below the limit of its detection [14].

## 3. Conclusions

Thus, as a result of the study, we have the following conclusions:

1. Hydrogel material “Colegel”, based on sodium alginate biopolymer, is non-toxic and does not cause inflammatory changes in tissues, which proves its safe use.

2. The optimal dose of amiodarone for local application to the atrial myocardium is a dose of 1 mg per 1 kg of body weight. At this dose, the clinically significant effectiveness of the amiodarone drug itself is determined and there are no violations of atrial and atrioventricular heart conduction.

3. The local use of hydrogel with amiodarone is clinically effective for preventing the development of POAF during CABG operations in patients with CHD. The use of Colegel hydrogel with amiodarone at a dose of 1 mg per 1 kg of body weight reduced the probability of developing POAF in the study group 18.9-fold.

## 4. Experimental Part

The experimental study on animals was carried out in the laboratory designed for modeling and studying the pathologies of the heart and vessels of the experimental Department of the Bakulev Scientific Center for Cardiovascular Surgery of the Russian Ministry of Health, in accordance with the requirements of the Local Ethical Committee (Protocol № 3 dated 08 February 2017). The animals were kept on a diet (P5025892) in compliance with the international recommendations of the European Convention for the Protection of Vertebrates Used for Experimental Purposes.

### 4.1. Hydrogel

We used a hydrogel material made on the basis of a biopolymer of sodium alginate and the drug amiodarone (the viscosity of the polymer composition was 4500 SP at a speed of 20 rpm; the consistency index was 27,310 SP; and the yield strength was −110.5 D/cml).

### 4.2. Experiment

The study was carried out on 46 rabbits of both sexes, weighing about 3–4 kg. Initially, the epicardial application of hydrogel material with amiodarone was performed on 36 rabbits. The animals for the experiment were divided into 5 groups:

- Group 1 (*n* = 8)—the dose of amiodarone in hydrogel was 1 mg;
- Group 2 (*n* = 11)—the dose of amiodarone in the hydrogel was 3 mg;
- Group 3 (*n* = 7)—the dose of amiodarone in the hydrogel was 6 mg;
- Group 4 (*n* = 10) control group—the application of the hydrogel material without amiodarone;
- Group 5 (*n* = 10)—group with an intravenous form of amiodarone at the dose of 60 mg.

Pericardiotomy was performed on the animals of this group without inserting anything into the cavity. Amiodarone was administered intravenously during the operation.

All animals underwent an atropine test (at the dose of 50 micrograms) to increase their heart rate (HR) (AF modeling) before and immediately after the operation, as well as before the control measurements 2 h after the operation, 1 and 3 days later. The invasive blood pressure, heart rate monitoring and ECG, indicators of inflammation according to laboratory tests and pathomorphological research, were evaluated. The animals from each group were removed from the experiment for the pathomorphological study of the heart on the 3rd, 7th and 14th days.

## 5. Clinical Part

The clinical stage of the study consisted of the randomized blind trial which included 60 patients who underwent CABG surgery. All patients were randomized into two groups: the study group (*n* = 30, with amiodarone hydrogel application) and the control group (*n* = 30, without hydrogel application). The dose of amiodarone in the hydrogel material was 1 mg per 1 kg of patient weight.

The exclusion criteria from the study were previous AF at baseline, CABG and the correction of the valvular heart apparatus, malignant neoplasms, severe renal failure, hormonal diseases, hyper- or hypofunction of the thyroid gland, and patients receiving immunosuppressive and anti-inflammatory therapy with a concomitant pathology.

All patients received optimal medical therapy and no specific antiarrhythmic therapy was prescribed before surgery.

### Surgery

CABG was performed in a standard way for our clinic. The operating access was performed through median sternotomy. The pericardium was opened in a longitudinal T-shaped way. Standard separate aortic cannulation, the separate cannulation of superior vena cava and inferior vena cava were performed. Artificial blood circulation was performed under normothermic conditions. Subsequently, the main stage of coronary artery bypass surgery was performed. After the end of the main stage of the operation, two epicardial electrodes were sutured to the right ventricle and two to the right atrium in all patients. Then, after the end of artificial blood circulation, the decannulation of the hollow veins and aorta was performed. The next step was the biatrial epicardial application of amiodarone hydrogel. The application of amiodarone hydrogel was performed using the 4 mL drug spray on the surface of both atria. The wounds were sutured layer by layer at the end of the operation.

## Figures and Tables

**Table 1:** Parameters of the studied rabbits.

Parameters	Group 1 (*n* = 8)	Group 2 (*n* = 11)	Group 3 (*n* = 7)	Group 4 (*n* = 10)	Group 5 (*n* = 10)	*p*
Aspartate aminotransferases (e/L)	33 (29.5; 35)	36 ± 6	33 ± 4	36 ± 6	31 ± 5	0.449
Alanine transaminase (e/L)	32.5 ± 6.4	34 ± 7	34 ± 1	34 ± 6	33 ± 5	0.689
WBC, ×10^9^/mL	5.8 ± 0.9	5.7 ± 1	5.2 ± 1.2	5.6 ± 0.8	6.4 (5.4; 6.7)	0.518
Neutrophils, ×10^9^/mL	63.7 ± 7.5	63 ± 4	64 ± 5	62 ± 5	58.5 (56; 66)	0.477
PQ (ms)	71.2 ± 8.3	70 ± 9	70 (60; 90)	73 ± 10	77 ± 9	0.801
QRS (ms)	60 (50; 60)	50 (50; 75)	50 (50; 70)	60 (50; 70)	62 ± 9	0.759
QT (ms)	135.6 ± 9	139 ± 14	130 ± 13	140 ± 11	141 ± 11	0.321
Hb (g/L)	141 ± 7	141 ± 8	137 ± 5.7	140 ± 8	139 (130; 149)	0.683
Procedure time, min	45 (43.5; 45.5)	42 ± 1	41 ± 3.7	44 ± 2	44 ± 2	0.014
APV time, min	35.2 ± 2.4	33 ± 2	35 ± 3.9	35 ± 2	36 ± 2	0.085

WBC, white blood cells; Hb, hemoglobin; APV, artificial lung ventilation.

**Table 2:** Dynamics of heart rate.

Heart Rate, *n*/min	Group 1	Group 2	Group 3	Group 4	Group 5	*p*
Before the procedure	145 ± 8	158 ± 16	153 ± 26	149 ± 14	154 ± 16	0.443
Before the procedure, after the atropine test	157.6 ± 6.2	170 ± 13	174 ± 29	165 ± 11	176 ± 12	0.285
After the procedure	145.5 (136; 151)	157 ± 19	90 (80; 100)	153 ± 17	149 ± 11	0.009
After the procedure, after the atropine test	152 (145; 162)	167 ± 18	95 (83; 105)	160 ± 18	153 (152; 160)	0.006
Two hours after the procedure, after atropine	149 (142; 153)	138 ± 12	89 ± 35	151 (147; 160)	155 ± 8	<0.001
One day after the procedure, after the atropine test	150 ± 21	131 (128; 144)	86 ± 27	167 ± 19	152 ± 9	<0.001
Three days after the procedure, after the atropine test	147 ± 18	130 ± 11	74 ± 13	162 ± 17	151 ± 12	<0.001

**Table 3:** Dynamics of some instrumental and laboratory parameters in groups 2, 3 and 5.

Parameters	Before the Procedure	After the Procedure	*p*
Group 2			
PQ, (ms)	70 ± 9	83 ± 9	0.002
QRS (ms)	50 (50; 75)	50 (50; 60)	0.169
QT, (ms)	139 ± 14	143 ± 13	0.137
AV-blockade 2–3	0	0	0.978
Aspartate aminotransferases (e/L)	36 ± 6	39 ± 9	0.318
Alanine transaminase (e/L)	34 ± 7	38 ± 7	0.092
Group 3			
PQ, (ms)	70 (60; 90)	90 (60; 100)	0.001
QRS (ms)	50 (50; 70)	80(60; 80)	0.063
QT, (ms)	130 ± 13	164 ± 12	<0.001
AV-blockade 2–3	0	71%	0.004
Aspartate Aminotransferases (e/L)	33 ± 4	35 (32; 60)	0.018
Alanine Transaminase (e/L)	34 ± 1	36 (32; 58)	0.020
Group 5			
PQ, (ms)	77 ± 9	85 (80; 90)	0.109
QRS (ms)	62 ± 9	62 ± 9	0.990
QT, (ms)	141 ± 11	148 ± 15	0.045
AV-blockade 2–3	0	20%	0.168
Aspartate aminotransferases (e/L)	31 ± 5	51 ± 21	0.039
Alanine transaminase (e/L)	33 ± 5	53 ± 26	0.037
AV, atrioventricular.			

**Table 4:** Clinical and instrumental parameters of the groups.

Parameters	Study Group (*n* = 30)	Control Group (*n* = 30)	*p*
Clinical data			
Age, years	61 ± 8.2	63 ± 8.8	0.264
Male, %	90	77	0.375
BMI	27.6 ± 3.5	29 ± 4.1	0.139
Prior MI, %	53	56	0.824
Remoteness of prior MI, month	7 (4; 18)	12 (7; 36)	0.117
Angina, CCS class	3 (3; 3)	3 (3; 3)	0.505
Stroke, %	0	6	0.657
Arterial hypertension, %	93	93	0.999
Smoking, %	27	20	0.657
Diabetes, %	30	10	0.183
COPD, %	23	13	0.505
Drug therapy			
ACEi	97	100	0.824
ASA	100	100	0.999
Statins	100	100	0.999
β-blockers	100	100	0.997
CCB	20	26	0.657
Instrumental data			
EDV, mL	111 (102; 129)	118 (107; 126)	0.584
ESV, mL	48 (41; 58)	47 (37; 58)	0.468
EDD, sm	5 (4.8; 5.2)	5 (4.9; 5.4)	0.217
LV EF, %	57 (56; 58)	58 (56; 59)	0.355
Left atrium volume, ml	79 (77; 80)	77 (69; 79)	0.039
Average heart rate (Holter ECG), *n*/min	68 (64; 70)	61 (59; 68)	0.060
PQ, ms	0.12 (0.12; 0.14)	0.12 (0.12; 0.14)	0.554
QT, ms	0.34 (0.32; 0.38)	0.34 (0.32; 0.36)	0.888
Laboratory data			
Creatinine, µmol/L	74 (70; 90)	84 (73; 102)	0.103
Glucose, mmol/L	5 (4.6; 6)	5 (4.5; 6)	0.778
WBC, *n* × 10^9^/mL	7 (6.3; 8.4)	7.6 (6; 8.3)	0.841
Fibrinogen, g/L	3 (2; 3)	3 (2; 3)	0.180

Note: BMI, body mass index; MI, myocardial infarction; CCS class of angina, Canadian Cardiovascular Society angina class; COPD, chronic obstructive pulmonary disease; ACEi, angiotensin-converting enzyme inhibitor; ASA, acetylsalicylic acid; CCB, calcium channel blockers; LA, left atrium; EDV, end-diastolic volume; ESV, end systolic volume; EDD, end diastolic dimension; LV EF, left ventricular ejection fraction; WBC, white blood cells.

**Table 5:** Intraoperative and postoperative parameters of the groups.

Parameters	Study Group (*n* = 30)	Control Group (*n* = 30)	*p*
Intraoperative parameters			
Operation time, h	3.9 ± 0.99	3.5 ± 0.85	0.379
Time of CPB, min	84 (65; 105)	75 (60; 97)	0.437
The number of grafts, *n*	2 (2; 3)	2 (2; 3)	0.657
Lactate (the end of the operation)	1.4 (1.2; 1.9)	1.5 (1.3; 2)	0.689
Glucose (the end of the operation)	7.3 (6.9; 9.4)	7 (6; 9.6)	0.610
Postoperative parameters			
Time of artificial lung ventilation, h	14 (12; 16)	16 (14; 16)	0.255
WBC, day 1	112 (10; 14)	13 (9; 13)	0.344
AF after surgery, %	3.3	37	<0.001
Lactate, day 5	0.6 (0; 1)	0.5 (0; 1)	0.898
WBC, day 5	11 (8.8; 12)	11 (9; 13)	0.270
Glucose, day 5	5 (4.9; 6)	5 (4.7; 5.7)	0.614
Creatinine, day 5	78 (70; 89)	79 (72; 110)	0.128
QT, day 5, ms	0.34 (0.32; 0.36)	0.34 (0.32; 0.36)	0.851
PQ, day 5, ms	0.14 (0.12; 0.16)	0.12 (0.12; 0.14)	0.002
Average heart rate (Holter ECG), day 5, *n*/min	59 (52; 60)	69 (65; 75)	<0.001
Minimum heart rate (Holter ECG), day 5, *n*/min	50 (49; 55)	55 (50; 58)	0.008
Number of bed-days	6 (6; 7)	8 (8; 9)	<0.001

Note: CPB, cardiopulmonary bypass; WBC, white blood cells.

**Table 6:** Cox regression model for assessing the risk of development of postoperative atrial fibrillation (POAF) in patients after coronary artery bypass grafting (CABG) (χ2 = 23.4; *p* = 0.0014).

Parameter	Regression Coefficient β	Standard Error	The Exponent Beta (Risk Index Exp, B)	Wald Criterion	*p*
Age, years	0.164	0.063	1.178	6.716	0.009
Application of amiodarone–hydrogel	−2.939	1.168	18.914	6.329	0.011
Time of CPB, min.	0.021	0.011	1.020	2.995	0.835
LV EF, %	−0.111	0.075	0.894	2.178	0.139
The number of grafts, *n*	−1.307	0.926	0.270	1.989	0.158
EDV, mL	−0.009	0.014	0.991	0.348	0.554
Male	−0.481	0.818	0.618	0.345	0.557

Note: CPB, cardiopulmonary bypass; LV EF, left ventricular ejection fraction; EDV, end diastolic volume.
